# Remote assessments of sleep and cognition in cognitively normal older adults at risk for Alzheimer disease

**DOI:** 10.1016/j.sleep.2025.108734

**Published:** 2025-12-19

**Authors:** Hannah M. Wilks, Matthew S. Welhaf, Andrew J. Aschenbrenner, Brian D. Carpenter, Brian A. Gordon, Suzanne E. Schindler, Brendan P. Lucey, John C. Morris, Jason Hassenstab

**Affiliations:** a Department of Psychological & Brain Sciences, Washington University in St. Louis, St. Louis, MO, USA; b Department of Neurology, Washington University in St. Louis School of Medicine, St. Louis, MO, USA; c Department of Radiology, Washington University in St. Louis School of Medicine, St. Louis, MO, USA; d Department of Psychology, North Carolina A&T State University, Greensboro, NC, USA

**Keywords:** Alzheimer’s disease, Sleep, APOE, AD biomarkers, Older adults, Ecological momentary assessments, Repeated measures design

## Abstract

**Study objectives:**

Sleep is a marker of brain function that could potentially identify patients for early intervention by detecting cognitive change during the early asymptomatic stages of Alzheimer disease (AD). We examined the relationship between sleep and cognition in cognitively unimpaired older adults and evaluated whether the relationship was altered by AD risk factors.

**Methods:**

Cognitively unimpaired older adults (N = 319, age 54–97 years) were administered a sleep diary (1x/day) and brief cognitive assessments (4x/day) for seven consecutive days via a smartphone application. We evaluated if a previous night’s sleep predicts next-day cognition and if sleep averaged over a week predicts cognitive performance averaged over a week. Additional analyses included the effects of carrying an apolipoprotein *(APOE)* ε4 allele and cerebrospinal fluid biomarkers of AD pathology.

**Results:**

At the between-person level, no associations were observed between sleep and cognition. Within-person analyses revealed that deviations (both higher and lower scores) from an individual’s usual sleep pattern were associated poorer next day cognitive performance. Carriage of the *APOE* ε4 allele and AD biomarkers did not interact with sleep-cognition relationships.

**Conclusions:**

Remote, multi-day assessments of cognition and sleep revealed subtle non-linear associations between nightly sleep and next-day cognition in cognitively unimpaired older adults. Furthermore, the individualized nature of sleep-cognition relationships underscores the importance of maintaining consistent person-centered sleep health metrics to support cognitive function. Capturing latent AD-related changes in sleep and cognition among asymptomatic older adults may require repeated assessments across extended timeframes.

## 1. Introduction

The progression of Alzheimer disease (AD) is slow, developing over approximately 20 years before clinical symptom onset [1]. Alterations to sleep and cognition emerge gradually in the “preclinical phase” of AD when individuals are considered cognitively unimpaired, but these changes are difficult to detect [2,3]. The cognitive consequences of poor sleep have been well established across the lifespan, with the most common impairments in attention [4–6] and memory [7–9]. However, it is unclear if the cognitive consequences of poor sleep are altered in the preclinical phase of AD. Characterization of the short-term consequences of a single night of poor sleep may reveal that those with preclinical AD experience exacerbated cognitive impairments after a poor night of sleep compared to individuals without preclinical AD. Investigating the link between sleep and cognition in preclinical AD might reveal a crucial window for intervention by identifying periods when poor sleep adversely affects cognition. This line of work is critical, as sleep interventions may be most effective at the preclinical stage, yet progress is limited by the challenges of identifying AD before symptoms arise.

To better understand the sleep-cognition-AD relationship, we can characterize risk for AD in cognitively normal individuals by considering genetic factors and pathological burden. Studies can classify those who are apolipoprotein *(APOE)* ε4 allele carriers and those who have abnormalities in cerebrospinal fluid (CSF) concentrations of common AD biomarkers, such as amyloid-β 42 (Aβ42), total tau, and phosphorylated tau at position 181 (p-tau181). These two groups provide insights into the differential effects of genetic risk and pathology, aiding in the characterization of disease progression. However, the current literature is mostly composed of laboratory-based examination of sleep-cognition-AD relationships, which limits our understanding of the complex and dynamic real-world presentation of cognitive fluctuations in preclinical AD after a poor night of sleep.

One of the most cost-effective and accessible ways to assess sleep is through daily sleep diaries in which aspects of sleep from the previous night are assessed the following day. Through sleep diaries, it is possible to assess five main components of sleep: satisfaction, alertness, timing, efficiency, and duration (SATED) [10]. Using the SATED model, each sleep health dimension can be rated on a Likert scale and summed to create a sleep health composite score that provides a reliable and comprehensive measure of sleep. Better sleep, as defined by higher scores on a sleep health composite, has been associated with better cognition across the lifespan [11,12]. Further, sleep regularity, or the day-to-day consistency of sleep, has been associated with health and well-being, such that worse regularity is correlated with poorer health and well-being [13–15]. Therefore, day-to-day variability in a sleep health composite over a short span of time may serve as a multidimensional proxy for sleep irregularity and may represent AD-related sleep dysfunction. To our knowledge, a multi-dimensional sleep health composite derived from a daily sleep diary has not yet been extended into AD research.

Digital cognitive assessments are becoming more common and provide a low-cost option to monitor sleep with survey instruments. Digital devices like smartphones can therefore repeatedly measure sleep and cognition over extended periods in naturalistic environments. Repeated measurement provides opportunities for within-person analyses, which might be necessary given the subtle and transient changes to sleep and cognition in preclinical AD. This work leverages technology to characterize a critical period along the disease processes when older adults are still cognitively unimpaired by integrating remote cognitive testing with a multi-dimensional sleep health composite. Such an approach has the potential to support clinical trials in identifying at-risk individuals most likely to benefit from sleep-based interventions.

The present study aimed to achieve two main objectives: (1) to establish the relationship between sleep and cognition in cognitively unimpaired older adults and (2) to examine how risk for AD (i.e., the presence of the *APOE* ε4 allele or preclinical AD pathology, as evidenced by abnormal levels of AD biomarkers) alters the sleep–cognition relationship. We tested these objectives by examining the between-person associations between sleep and cognition across two time domains: a week and a single day. We hypothesized a positive linear relationship between sleep and cognition, such that higher scores on the sleep health composite would be associated with better cognition in cognitively unimpaired older adults. We further hypothesized that AD risk would interact with the linear sleep–cognition relationship, particularly when sleep was poor, such that lower scores on the sleep health composite would be associated with worse cognition in those with AD risk factors compared to those without such risk factors. To capture the nuances of within-person sleep–cognition associations, we examined whether nightly deviations from an individual’s typical sleep predicted next-day cognitive performance. We hypothesized that if participants’ prior night of sleep score was below their weekly average sleep health score, it would result in worse next-day cognition. We further hypothesized that the inverse would be true; when participants’ previous night’s sleep score was higher than their weekly average, it would predict better next-day cognition.

## 2. Materials and methods

### 2.1. Participants

Participants were enrolled from ongoing studies of aging and AD risk at the Charles F. and Joanne Knight Alzheimer’s Disease Research Center (Knight ADRC) at Washington University School of Medicine in Saint Louis. Knight ADRC participants were co-enrolled in the Ambulatory Research in Cognition (ARC) smartphone study beginning in early 2020. Data from March 2020 to January 2024 were included. All participants provided informed consent, and all procedures were approved by the Human Research Protections Office at Washington University in St. Louis.

### 2.2. Clinical assessments

The Clinical Dementia Rating^®^ (CDR^®^) was used to assess the presence and severity of dementia. The CDR is a semi-structured clinical interview that measures cognitive and functional deficits across six domains, including memory, orientation, judgment & problem-solving, community affairs, home & hobbies, and personal care [16]. There are five global CDR stages: 0, 0.5, 1, 2, and 3 (normal cognition, very mild dementia, mild dementia, moderate dementia, and severe dementia, respectively).

### 2.3. Smartphone assessments

#### 2.3.1. Cognitive measures

**Fig. 1.**
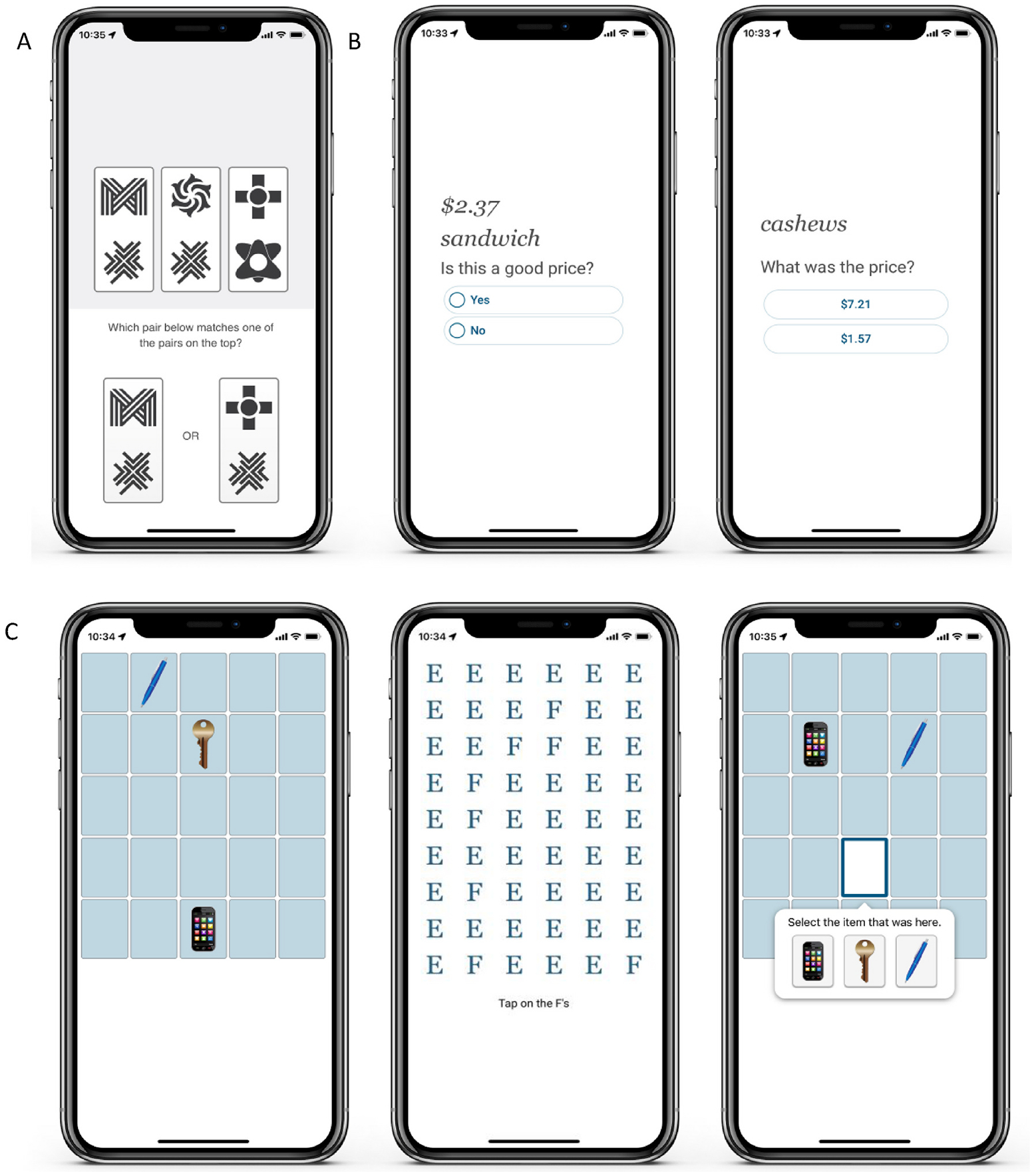
(A) Symbols (B) Prices (C) Grids.

Participants completed brief cognitive tests up to four times per day for seven consecutive days using the ARC smartphone application. A detailed description of the ARC platform has been published elsewhere [17]. The Symbols test is a processing speed task [18–20]. Participants are asked to identify an abstract shape pair on the top of the screen that matches one of the abstract shape pairs on the bottom of the screen. Participants complete 12 trials as quickly as possible. The dependent variable was the median response time (RT) of correct trials. The Prices test is an associative memory task that consists of a learning phase and a recognition phase [18,21]. Participants are shown common shopping items (e.g., “sandwich”) paired with a price (e.g., $2.37) during a learning phase. Ten item-price pairs are presented. During the recognition phase, participants are shown a studied shopping item and two prices and are required to select the price that corresponds to the one previously shown. The dependent variable is the error rate. The Grids test is a spatial working memory task. Participants are shown three common household items on a 5 x 5 grid during the learning phase. Following a visual search distraction task, participants are instructed to place the studied objects in the correct location. The dependent variable is the Euclidean distance of each item from their original location. Examples from the Symbols, Prices, and Grids tests are shown in Fig. 1.

A composite was created to summarize performance across ARC measures at both the daily and weekly levels. Each ARC measure was first standardized to the sample mean and standard deviation to create a z-score. Each z-score was then summed and averaged to produce an ARC cognitive composite for each session. For the analyses focused on examining the associations between previous night of sleep and next day cognition, the composite was calculated as the average across all sessions within a day. For the analyses focused on examining the associations between weekly sleep and cognition, the daily cognitive composite was then averaged across the week. In all cases, higher scores reflected worse performance.

#### 2.3.2. Sleep diary and alertness visual analog scale

**Fig. 2.**
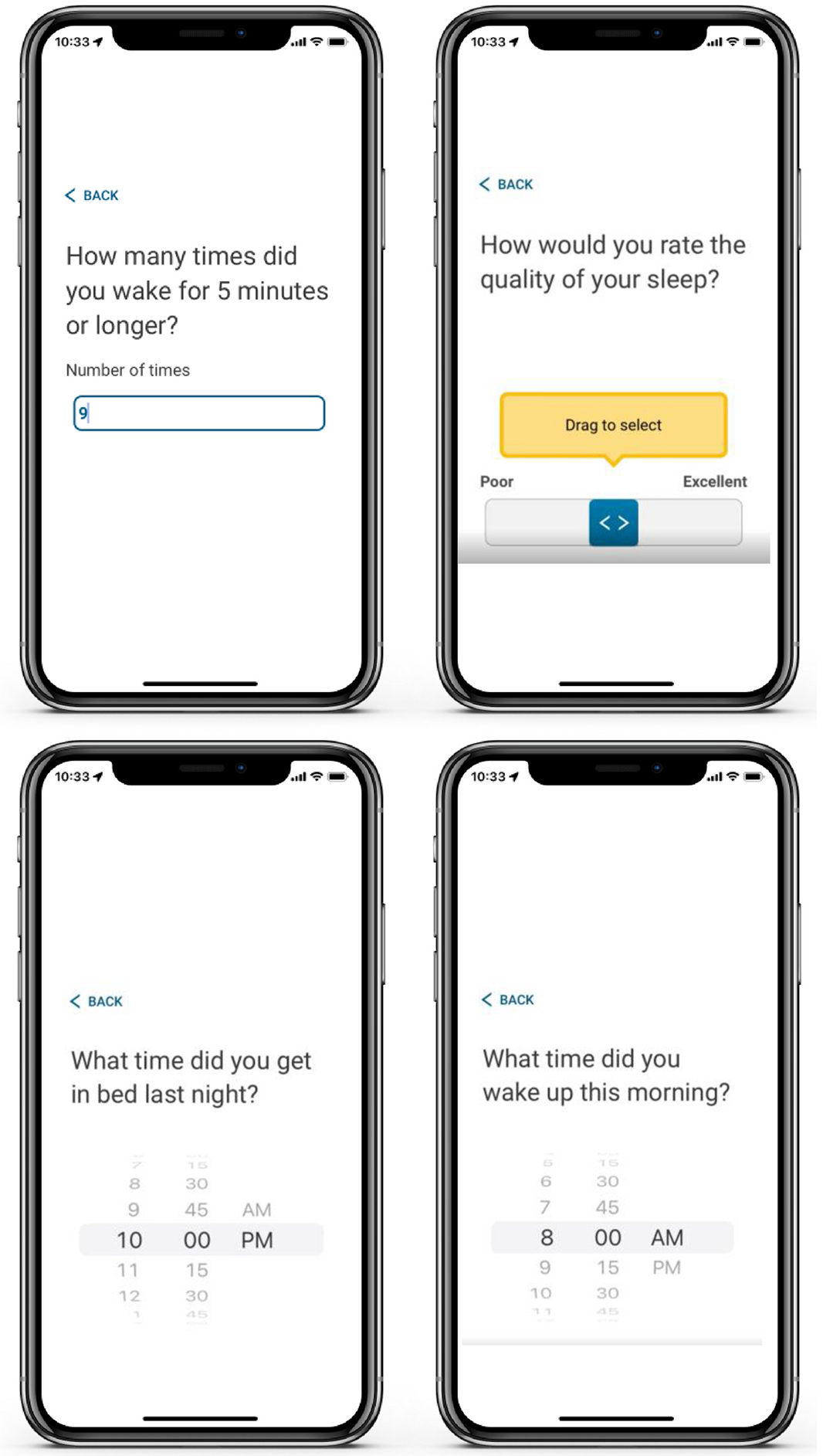
Visualizations of the daily sleep diary.

A sleep diary was administered each morning using the ARC smartphone application. The sleep diary queries for bedtime (“What time did you get in bed last night?”), sleep onset latency (“How long did it take you to fall asleep last night?”), nighttime awakenings (“How many times did you wake for 5 min or longer?”), waketime (“What time did you wake this morning?”), waketime latency (“What time did you get out of bed this morning?”), and sleep quality (“How would you rate the quality of your sleep?”). Sleep quality will be referred to as sleep satisfaction hereafter. A visualization of the sleep diary is included in Fig. 2.

**Fig. 3.**
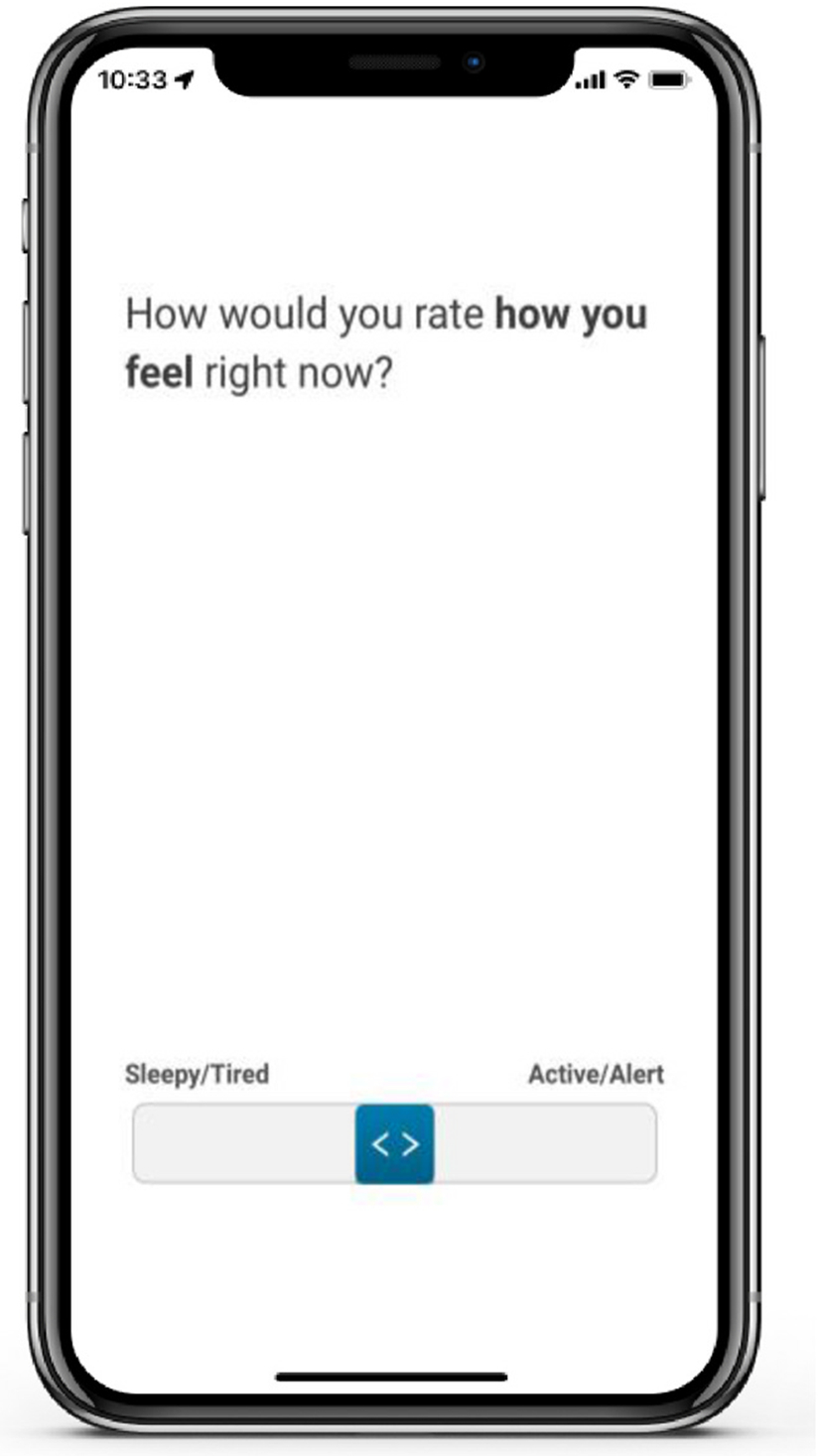
Visualization of the fatigue visual analog scale.

Alertness was assessed with a simple visual analog scale (VAS) each session. A visualization of the alertness VAS is included in Fig. 3. The alertness VAS queries about current energy level, ranging from *very sleepy/tired* to *very active/alert*. Scores range from 0 to 1, with lower scores indicating more fatigue. The dependent variables were daily average alertness and weekly average alertness.

#### 2.3.3. Sleep health composite

From the sleep diary and fatigue VAS, we constructed a multidimensional sleep health composite designed to capture key aspects of sleep: satisfaction, alertness, timing, efficiency, and duration. Our multidimensional sleep health composite was adapted from the 5-item sleep health scale [10]. Here, each component was dichotomized, with indicators of good sleep assigned a score of 1 and indicators of poor sleep assigned a score of 0, allowing us to generate a composite score reflecting overall sleep health. Where possible, we used the cutoffs from the original sleep health scale [10].

**Fig. 4.**
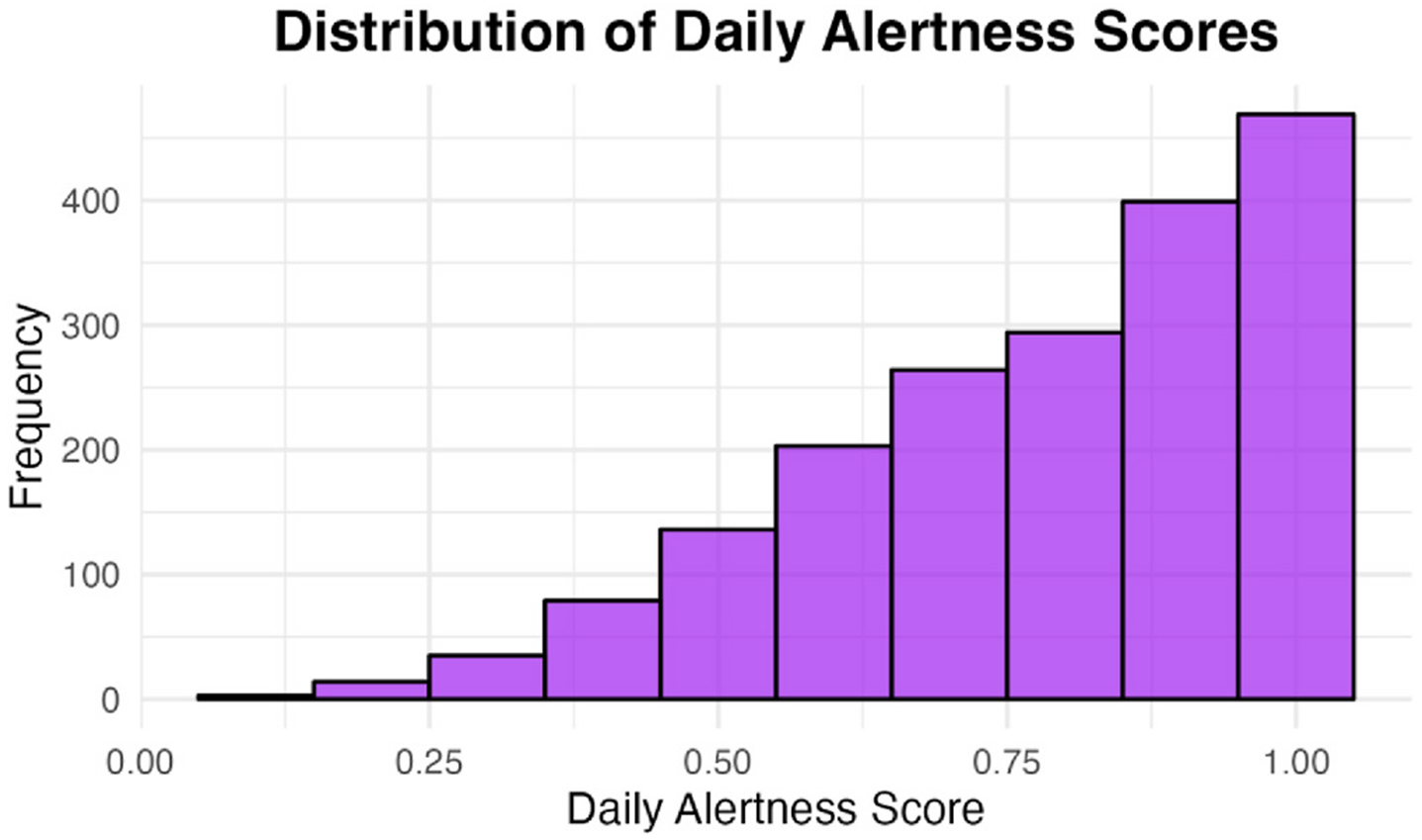
Distribution of daily alertness score (N Participants = 319). The visual analog scale ranged from 0 (sleepy/tired) to 1 (active/alert).

**Fig. 5.**
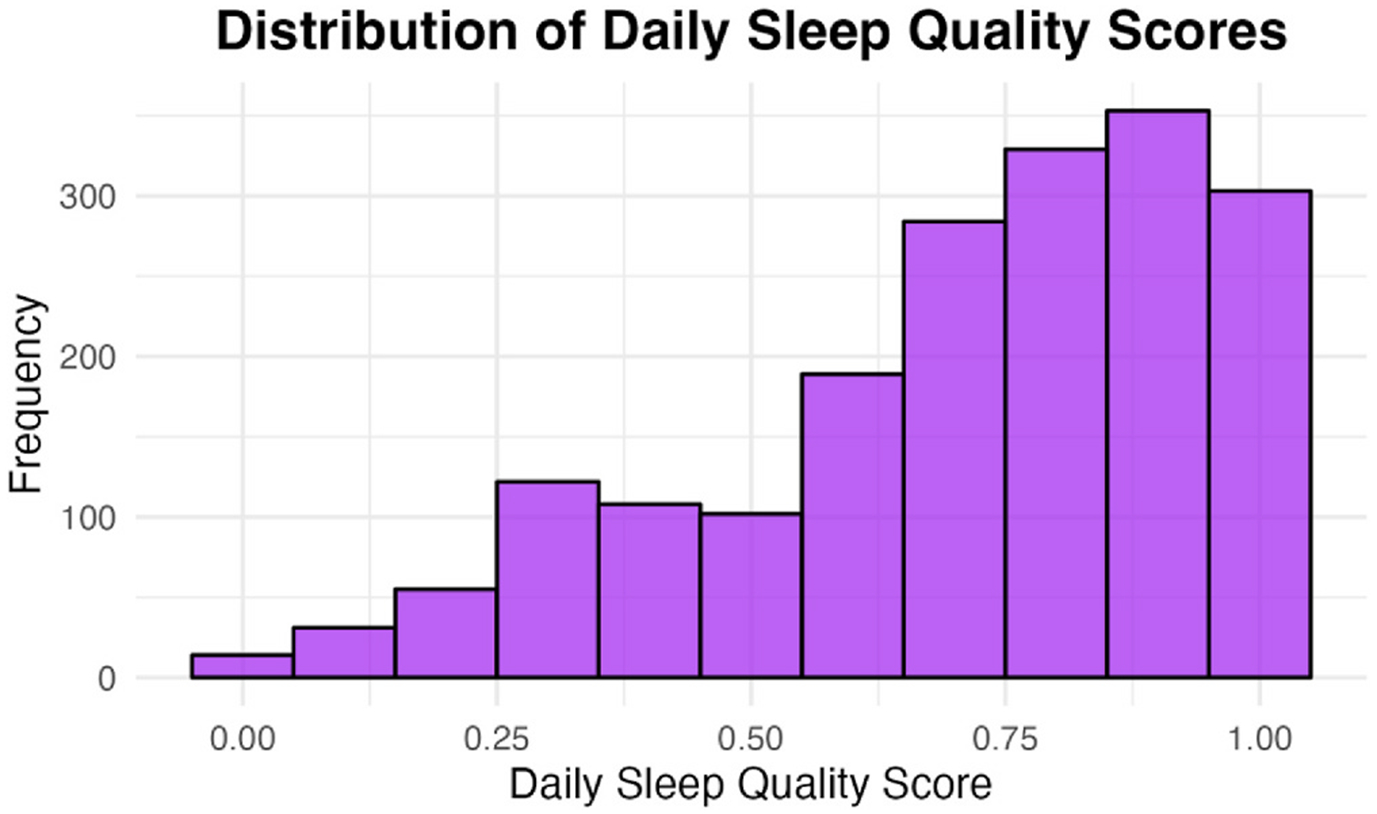
Distribution of daily sleep quality scores (N Participants = 319). The visual analog scale ranged from 0 (poor) to 1 (excellent).

Sleep satisfaction was measured using the sleep quality VAS, with scores greater than 0.8 (on a 0–1 scale) classified as indicative of good sleep satisfaction. Alertness was assessed via the alertness VAS, where scores above 0.8 similarly represented good alertness. Visual inspection of responses for satisfaction and fatigue revealed a notable response bias towards the highest rating (1), which may reflect participants’ tendencies to report generally positive sleep experiences or alertness levels (Figs. 4 and 5, respectively). A cutoff of 0.8 was selected in a data-driven approach, as it approximated the mean value for both items and offered a practical means of distinguishing higher-quality sleep experiences from more average or lower-quality responses. This approach allowed us to capture meaningful variation in perceived sleep quality and fatigue without relying on arbitrary thresholds or clinical judgment alone. Sleep duration was calculated using participants’ self-reported bedtime and wake time, from which the sum of sleep onset latency and the number of awakenings lasting 5 min or longer was subtracted. Durations between 6 and 8 h were classified as indicative of good sleep duration. This range was selected based on systematic reviews indicating that “short” sleep durations (i.e., less than 6 h) and “long” sleep (i.e., sleep durations greater than 8 h) were associated with poor health outcomes in older adults [22]. Sleep durations which included sleep between 2:00 and 4:00 a.m. was categorized as good sleep timing [10]. Efficiency was calculated as the sum of the number of awakenings that were 5 min or longer and sleep onset latency in minutes, with less than 30 min spent awake each night rated as good sleep efficiency [10]. See Table 1 for a summary of the sleep composite.

Three outcome measures were derived from the sleep health composite: a between-person daily composite, a between-person weekly composite, and a within-person daily deviation score. The daily sleep composite was the sum of the five sleep domains (satisfaction, alertness, timing, efficiency, and duration). The maximum daily score was five and reflected the best daily sleep health composite score. The weekly sleep composite was the average of the daily sleep composite across the week. Again, the maximum weekly score was five and reflected the best weekly sleep health composite score. To capture daily, within-person changes from typical sleep patterns, a deviation score was created reflecting the difference between the daily sleep composite and the weekly sleep composite. Positive scores reflected higher sleep health composite scores than average.

Dimensionality of the sleep composite was evaluated post hoc. The selected items were binary and exhibited low variance. As such, a confirmatory factor analysis did not converge. As shown in Table 2, Tetrachoric correlations showed two clusters of related items (i.e., Alertness and Satisfaction; Timing and Duration). Minimal variance and weak associations were observed with Efficiency. These results suggest that the composite reflects an index of related sleep characteristics rather than a single latent factor.

### 2.4. Genotyping and cerebrospinal fluid

Blood samples were drawn to determine *APOE* ε genotyping. Participants were classified as *APOE* ε4+ (ε4/ε4, ε3/ε4, ε2/ε4) or *APOE* ε4-(ε3/ε3, ε2/ε3, ε2/ε2). A more detailed description of the methods for obtaining *APOE* status has been described elsewhere [23].

Knight ADRC participants are invited to undergo a lumbar puncture to collect CSF approximately every three years. CSF data were collected using procedures described in detail previously [24]. Processed samples were measured by chemiluminescent enzyme immunoassay using LUMIPULSE G1200 (Fujirebio, Malvern, PA) as described in manufacturer instructions to obtain concentrations of Aβ42 and p-tau181. A ratio of p-tau181 to Aβ42 (p-tau181/Aβ42) >0.0649 was used as the indicator of AD pathology, as we have published previously [21].

### 2.5. Inclusion criteria

Participants were included if they could independently operate a smartphone and had no evidence of cognitive impairment, as indicated by a CDR of 0. Participants underwent the annual clinical assessment within approximately 55 days of completing the ARC assessment (SD = 44.8; Range = 1–259). To ensure appropriate adherence to ARC, participants needed to complete at least 10 ARC sessions across the week. To ensure that there were enough data points to compute the daily ARC composite, at least three of four daily ARC sessions needed to be completed for those values to be retained. Three hundred and thirty-six participants met these criteria, for a total of 2068 observations. Removal of erroneous sleep data, defined as entries missing AM/PM timestamps (92 sessions), incomplete surveys (18 sessions), negative sleep durations (105 sessions), or sleep durations greater than 16 h (62 sessions), further reduced the sample to three hundred and nineteen participants, for a total of 1791 observations.

A maximum of five years was permitted to elapse between CSF collection and ARC testing to maximize power. Recent research has demonstrated that only a small portion of those who were CSF amyloid negative convert to amyloid positive in this timeframe [25]. The slow progression of the changes in pathology and small likelihood of progression suggests that most of our participants who were classified as biomarker negative did not convert to biomarker positive. 203 participants had CSF data within five years of ARC testing, for a total of 1160 observations. CSF samples were collected on average 2.4 ± 1.4 years from the ARC testing.

### 2.6. Statistical analysis

Statistical analyses were completed using R 4.3.2 statistical software [26]. Linear regression models determined the relationship between cognition and sleep averaged at the weekly level. Models included terms for age (z-scored), self-reported gender (dummy coded with male as the reference group), and years of education (z-scored). Linear mixed-effects models determined the relationship between cognition and sleep from the previous night. These models were conducted using the *lme4* package with degrees of freedom calculated with the Satterthwaite approximation [27]. Models included the same covariates listed above, in addition to the maximum number of days with at least 75 % of ARC tests completed and a random intercept across all participants. To evaluate whether a non-linear model better captured the relationship between nightly sleep deviations and next-day cognition, we compared a linear model to a generalized additive mixed model (GAMM) that included a non-linear smoothing term for sleep deviation. The GAMM with the smoothing term demonstrated a lower Akaike Information Criterion (AIC) value compared to the linear model, indicating improved model fit. As such, the non-linear model was selected for subsequent analyses. These models were conducted using the *mcgv* package [28]. Models included the same covariates as those described above in the linear regression models, except a spline fit was applied for the sleep composite deviation and individual random effect. The model’s distribution was assumed to be Gaussian, and Restricted Maximum Likelihood was used to estimate the model parameters.

Models included additional terms for *APOE* ε4 status (carrier/non-carrier) and CSF p-tau181/Aβ42 status (positive/negative), where appropriate. Due to the high collinearity between AD biomarker positivity and *APOE* ε4 status, *APOE* ε4 status was removed as a term in models that included CSF p-tau181/Aβ42 status.

Analyses began by determining whether sleep had a main effect on cognition over and above the effects of the terms described above. Next, analyses considered whether *APOE* ε4 status interacted with the association between sleep and cognition. Finally, the subset of the sample with CSF p-tau181/Aβ42 status was used to determine if p-tau181/Aβ42 status interacted with the association between sleep and cognition.

## 3. Results

### 3.1. Demographics and descriptives

**Fig. 6.**
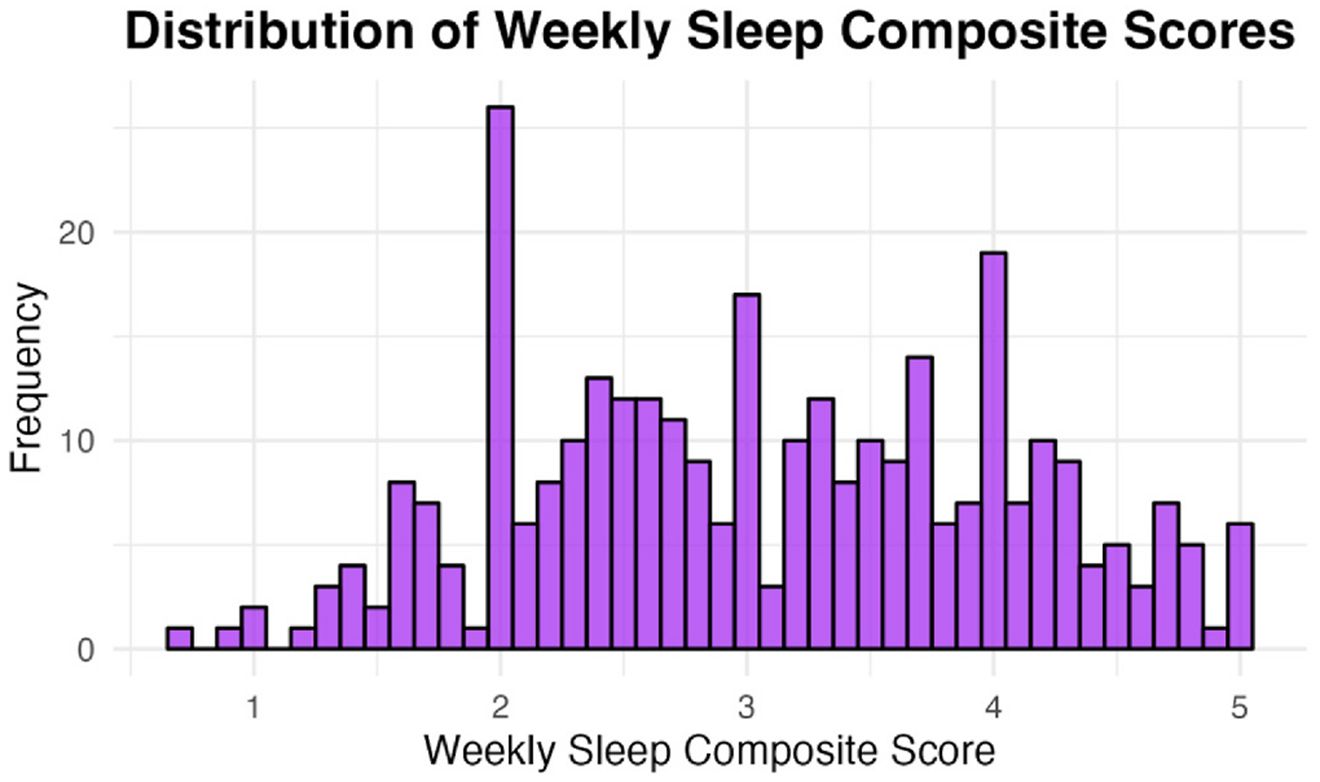
Histogram of the weekly sleep composite scores (N = 319).

Sample demographics by biomarker status are described in Table 3. Of the 319 participants, 83.7 % (N = 267) of the sample self-reported their race as White. The mean age was 75.8 years (SD = 5.9, Range = 54–97). The sample was highly educated (Mean = 16.4 years, SD = 2.5) and a majority self-reported their gender as female (N = 190, 59.6 %). Approximately one-third of the sample carried an *APOE* ε4 allele (N = 115, 36.2 %). The ARC composite was 0.1 across the week (SD = 0.7). When averaged across a week of ARC testing, participants’ weekly sleep composite was 3.1 (SD = 1.0, Range 0.8–5.0, Fig. 6).

In the subset of our sample with CSF biomarker data (N = 203), 30 % of participants were categorized as p-tau181/Aβ42 positive (N = 59). Participants with AD biomarker pathology were older (*p* = 0.002), more likely to carry an *APOE* ε4 allele (*p* < 0.001), and had worse cognition (*p* < 0.001) than those who were biomarker negative. There were no differences by self-reported gender, race, or education.

Individual sleep composite variables at the daily level by CSF biomarker status are also described in Table 3. Most participants were asleep between 2 and 4 a.m. (96.5 %). More variability was observed for satisfaction, alertness, efficiency, and sleep duration, with 43.5 % meeting the criteria for high satisfaction, 54.7 % meeting the criteria for high alertness, 58.8 % reporting less than 30 min spent awake each night, and 51.5 % with sleep durations between six and 8 h. Individual sleep composite variables at the daily and weekly levels did not differ by AD pathology (all *p*’s > 0.05).

### 3.2. Weekly between-person Sleep–Cognition associations

We first examined whether sleep predicted cognition when averaged across the week. The output of the models can be seen in Table 4. Weekly cognition was significantly predicted by age (all *p*’s < 0.001) and education (Model 1 *p* = 0.044). No associations were observed between sleep and cognition when averaged across a week (*p* > 0.05). Further, risk factors for AD did not interact with the association between weekly sleep and weekly cognition (all *p*’s > 0.05).

### 3.3. Daily between-person Sleep–Cognition associations

We then investigated whether the prior night of sleep predicted next-day cognition. As seen in Table 5, daily cognition was significantly predicted by age (all *p*’s < 0.001), education (Model 1 *p* = 0.018 and Model 2 *p* = 0.035), the maximum number of days with at least 75 % of ARC tests completed (Model 1 *p* = 0.001, Model 2 *p* = 0.002, Model 3 *p* = 0.002), and *APOE* ε4 allele status (Model 2 *p* = 0.004). The previous night of sleep did not predict next-day cognition (*p* > 0.05). Carriage of the *APOE* ε4 allele and CSF p-tau181/Aβ42 status did not interact with the previous night’s sleep on next-day cognition (p’s > 0.05).

### 3.4. Daily within-person Sleep–Cognition associations

**Fig. 7.**
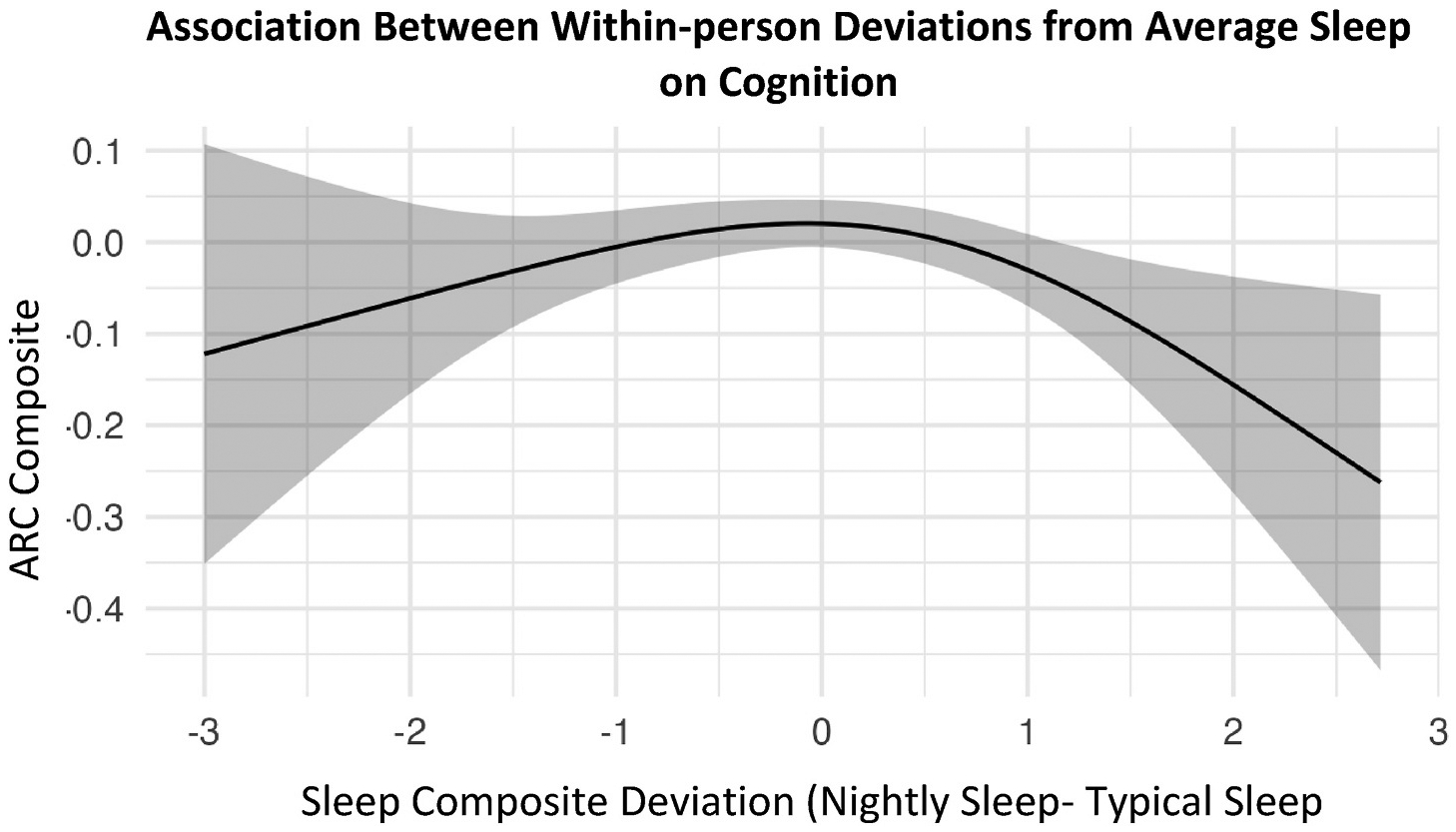
Within-person deviations from average sleep were associated with worse cognition (N = 319). Higher scores for the within-person deviations from the sleep composite indicate better sleep health than average. For visualization purposes, the ARC composite scores were inverted (multiplied by −1) so that higher values on the y-axis reflect better cognitive performance.

We then used generalized additive models to examine whether daily, within-person changes from average sleep patterns predicted next-day cognition (Table 6). Cognition was significantly predicted by age (all *p*’s < 0.001), gender (Model 1 *p* = 0.005), education (Model 1 *p* < 0.001 and Model 2 *p* < 0.002), the maximum number of days with at least 75 % of ARC tests completed (all *p*’s < 0.001), carriage of the *APOE* ε4 allele status (Model 2 *p* < 0.001) and CSF p-tau181/Aβ42 status (Model 3 *p* < 0.001). Sleep composite deviation was found to have a significant spline fit. In other words, higher and lower scores on the sleep health composite, compared to an individual’s average sleep health scores, were associated with worse next-day cognition (t (2.7) = 3.5, *p* = 0.031, Fig. 7). Carriage of the *APOE* ε4 allele and CSF p-tau181/Aβ42 status did not interact with the sleep composite deviation on next-day cognition in generalized additive models (all *p*’s > 0.05).

## 4. Discussion

In this study, we examined associations between sleep and cognition in a well-characterized sample of cognitively unimpaired older adults at risk for AD. Our hypotheses were partially supported — we found that better and worse scores on a sleep health composite, when compared to an individual’s average scores on the sleep health composite, were associated with worse next-day cognition. These results provide evidence that daily sleep variability in either direction is predictive of subtle declines in next-day cognition in cognitively normal older adults. In our study, scores on a five-item sleep health composite did not differ by AD risk factors. Additionally, AD risk factors did not moderate the association between sleep and cognition. These results suggest that sleep health and day-to-day cognition may be relatively stable across biomarker and genetic risk groups in cognitively unimpaired older adults.

The first major finding was that nightly changes in typical sleep patterns were associated with worse next-day cognition in cognitively unimpaired older adults. Notably, worse next-day cognition was observed not only when scores on the sleep health composite were below the individual’s typical score, but also when they were above it. Furthermore, the daily sleep composite alone was not predictive of next-day cognition. These findings suggest that within-person fluctuations in sleep may be more consequential for cognitive performance than between-person differences, and that maintaining consistent sleep health may be more important than achieving an optimal sleep health score. This underscores the value of within-person approaches in sleep and cognition research, as traditional between-person designs may miss individualized, dynamic changes that precede clinical symptoms [20]. Monitoring these fluctuations over time may improve the characterization of AD.

Prior work supports this interpretation, highlighting sleep regularity as a strong predictor of health and underscoring the non-linear nature of the sleep-cognition relationship [13–15]. However, such patterns may have been obscured in the existing literature. Consistent sleep durations have been used as a proxy for sleep regularity [29–32]; however, other key dimensions of sleep health, such as nighttime awakenings, sleep quality, and sleep onset latency, are often neglected. We uniquely defined “regularity” as the change in the sleep health composite, as opposed to defining regularity based on sleep duration. A strength of this approach was that we were able to maintain the multi-dimensional nature of the sleep health composite and unmask how day-to-day fluctuations across several aspects of sleep jointly contribute to overall sleep regularity. This approach moves beyond duration-focused definitions of regularity and captures a more holistic profile of sleep health, which may be particularly important in aging populations where disruptions in multiple sleep domains often co-occur. Future research should investigate sleep regularity using multidimensional measures to better identify patterns that may influence cognitive outcomes in older adults.

Our second main finding came from analyses focused on how risk factors for AD alter the relationship between sleep and cognition in cognitively unimpaired older adults. We assessed AD risk in two ways: by genetic risk (*APOE* ε4 carriage) and by classifying participants as having preclinical AD as indicated by the presence of abnormal biomarkers without cognitive impairment. In both cases, AD risk did not significantly modify the association between sleep and cognition. This was surprising, given that sleep changes are common in *APOE* ε4 carriers [33–35] and in preclinical AD [36]. A recent study by Fenton and colleagues [37] examined associations between cognition and objectively measured, within-person variability for sleep duration and efficiency. Further, they explored the modifying effects of AD risk factors on the sleep-cognition relationship. They reported that greater variability in sleep duration and efficiency was associated with worse cognition and greater *β*-amyloid burden. Notably, *APOE* ε4 carriers benefited from more consistent sleep and longer sleep durations, as evidenced by less *β*-amyloid burden and better cognition. Similar findings were reported for Lim and colleagues [38], such that *APOE* ε4 carriers who were “good” sleepers had a decreased risk of progressing to AD dementia. We suspect that differences in study design contribute to our differing results. First, the present study enrolled only cognitively normal older adults, whereas Fenton and colleagues included older adults with symptomatic cognitive decline, potentially increasing their ability to detect associations between *β*-amyloid burden, cognition, and sleep in *APOE* ε4 carriers. Second, the present study assessed sleep via a sleep diary instead of a physical activity monitor, which Fenton and colleagues used. Their use of objective sleep measurement may have further increased their ability to detect an effect, as it did not rely on retrospective self-reports of sleep. Another potential explanation for our dissimilar findings may be that reducing recall biases through a daily sleep diary and transforming the sleep diary data into a 5-point sleep health composite was not enough to overcome the challenges associated with self-reported sleep in the preclinical stages of AD. Future research should investigate whether incorporating additional components theorized to reflect sleep health, such as consistent bedtimes and waketimes, may be necessary to unmask subtle sleep–cognition changes in preclinical AD.

Lastly, the lack of a significant interaction between AD risk factors, sleep, and cognition could be attributed to the characteristics of our sample, which consists of mostly white and highly educated participants from the United States. Multiple studies have reported that minoritized group status may interact with the association between sleep and cognition [39,40]. Further, self-reported sleep parameters have been shown to differ by economic status [31], and it is unclear whether economic status may interact with the sleep composite used in the present study. Finally, the participants self-selected to join the present research study, which required participants to complete (1) an annual clinical and cognitive assessment and (2) cognitive testing on the ARC application. While efforts were made to increase the accessibility of the ARC platform [41], the present sample likely reflects a unique subgroup of older adults who were willing and able to participate in the study.

A key strength of the study is the use of high-frequency, smartphone-based cognitive assessments and daily sleep diaries, which enabled the examination of both general sleep-cognition patterns and acute, within-person changes from day to day. We created a five-item multidimensional sleep composite that captured several dimensions of sleep health, allowing us to holistically represent an individual’s sleep health. While this composite did not predict cognition independently, it allowed us to isolate the unique impact of nightly deviations in sleep. Further, we incorporated a powerful modeling approach with GAMMs to better understand the non-linear association between sleep and cognition. This analytic approach avoids assumptions of linearity, which is especially important given evidence that the sleep-cognition relationship is complex and potentially non-linear. Together, this multidimensional and data-intensive design allowed us to move beyond traditional between-person comparisons and contribute foundational evidence on within-person associations between sleep and cognition in cognitively unim-paired older adults. Finally, our sample was well characterized. This enabled us to test whether AD risk factors at multiple time points before symptom onset (i.e., genetic risk for AD and AD biomarker abnormalities) altered the relationship between sleep and cognition, and to determine whether these risk factors magnified subtle associations that might otherwise go undetected. By incorporating these complementary markers of AD risk, we were able to rigorously evaluate the robustness of sleep–cognition associations in the preclinical stage.

In summary, we developed a multi-dimensional sleep composite using data from a brief, multifaceted sleep diary administered remotely through a smartphone-based platform. This approach, combined with high-frequency cognitive measurements, provided a unique opportunity to test the relationship between daily changes in self-reported sleep and daily cognitive fluctuations in preclinical AD. Our findings indicate that cognition is sensitive to nightly variations in self-reported sleep, especially when evaluated using a multidimensional approach. This research underscores the importance of repeated monitoring of self-reported sleep and cognition in older adults. Future studies should aim to monitor self-reported sleep and cognition over several months or even years in participants’ naturalistic environments to detect more subtle changes over time. Such prolonged and continuous monitoring could serve as a non-invasive marker for AD, which could be invaluable in the design and implementation of sleep-based clinical trials aimed at preventing or slowing the progression of the disease. Moreover, if future work confirms that irregular sleep patterns contribute to cognitive vulnerability and the development of AD, this line of research could inform individualized “prescriptions” for sleep health by focusing not only on sleep duration and efficiency, but also on maintaining regular sleep-wake schedules. Such insights could, in turn, support the development of behavioral interventions or real-time digital feedback tools designed to help older adults adopt and sustain consistent sleep routines that promote healthy aging.

## Figures and Tables

**Table 1:** Sleep Composite. Five domains were selected from questions on the ARC application. Satisfaction, Timing, Efficiency, and Duration were determined from the daily sleep diary. Alertness was assessed before each ARC cognitive testing session. Alertness scores were averaged across the day to represent daily alertness. Scores on each domain were added to create a sleep composite where numbers better

Variables		0 (Poor Sleep)	1 (Good Sleep)
**Satisfaction**	How would you rate the quality of your sleep? (0–1)	Less than 0.8	Greater than 0.8
**Alertness**	How alert are you? (0–1)	Less than 0.8	Greater than 0.8
**Timing**	Asleep between 2:00 and 4:00 a.m.	No	Yes
**Efficiency**	Less than 30 min awake each night, including sleep onset latency and nighttime awakenings greater than 5 min	No	Yes
**Duration**	Sleep duration between 6 and 8 h	No	Yes

**Table 2:** Sleep Composite Tetrachoric Matrix. For validation, tetrachoric correlations were used to determine the structure of the five items comprising the sleep health composite. Two clusters of related variables emerged (Satisfaction + Alertness; Duration + Timing), while Efficiency showed minimal variance and weak associations with the other variables.

	Satisfaction	Alertness	Efficiency	Duration	Timing
**Satisfaction**	1.00	0.62	0.35	0.03	0.24
**Alertness**	0.62	1.00	0.18	0.05	0.15
**Efficiency**	0.35	0.18	1.00	−0.01	−0.08
**Duration**	0.03	0.05	−0.01	1.00	0.45
**Timing**	0.24	0.15	−0.08	0.45	1.00

**Table 3:** Demographics, cognition, and sleep by CSF biomarker status. Statistical Differences were calculated using Wilcoxon rank tests and chi-squared tests for continuous and categorical variables, respectively. A *p*-value compares the CSF- positive and CSF-negative groups. CSF positivity was defined as CSF p-tau181/ Aβ42 *>* 0.0649.

Variables	All Participants (N = 319)	CSF p-tau181/Aβ42 Positive (N = 59)	CSF p-tau181/Aβ42 Negative (N = 144)	*p*-value CSF Positive vs Negative
Age (mean years, SD)	75.8 (5.9)	76.9 (5.6)	74.5 (5.8)	0.002
Gender (n, % female)	190 (59.6)	27 (54.2)	86 (54.2)	1
Race (n, %)				0.580
Black	51 (16.0)	4 (6.8)	15 (10.4)	
White	267 (83.7)	55 (93.2)	128 (88.9)	
More than one	1 (0.3)	0 (0.0)	1 (0.70)	
Education (mean years, SD)	16.4 (2.5)	16.3 (2.6)	16.6 (2.2)	0.459
*APOE* ε4 Carrier Status (n, % Positive)	115 (36.2)	37 (62.7)	36 (25.0)	<0.001
Weekly ARC Cognitive Composite (mean, SD)	0.1 (0.7)	0.2 (0.5)	−0.01 (0.64)	<0.001
Weekly ARC Sleep Composite (mean, SD)	3.1 (1.0)	3.1 (1.0)	3.2 (1.0)	0.670
Daily Sleep Satisfaction (n, % good)	777 (43.5)	143 (42.3)	377 (46.5)	0.218
Daily Sleep Alertness (n, % good)	980 (54.7)	187 (55.3)	484 (59.5)	0.219
Daily Sleep Timing (n, % good)	1729 (96.5)	328 (97.0)	726 (97.5)	0.777
Daily Sleep Efficiency (n, % good)	1071 (58.8)	202 (59.8)	514 (63.1)	0.312
Daily Sleep Duration (n, % good)	924 (51.5)	176 (52.1)	420 (51.6)	0.935

**Table 4:** Linear regressions predicting weekly cognition. CSF positivity was defined as CSF p-tau181/Aβ42 *>* 0.0649.

Covariate	Model 1 – Full Sample	Model 2 – Full Sample; *APOE* ε4 Interaction	Model 3 - Subsample; CSF Biomarker Interaction
	*b (SE)*	*b (SE)*	*b (SE)*
Age (z-scored years)	0.16 (0.03) ***	0.18 (0.03) ***	0.17 (0.04) ***
Gender (Male)	0.10 (0.06)	0.07 (0.06)	0.10 (0.09)
Education (z-scored years)	0.06 (0.03) *	−0.05 (0.03)	0.03 (0.04)
Sleep Composite	0.01 (0.03)	−0.02 (0.04)	−0.02 (0.05)
*APOE* ε4 Status (Positive)		0.21 (0.21)	
Sleep Composite x *APOE* ε4 Status		0.01 (0.07)	
CSF Biomarker Status (Positive)			0.08 (0.31)
Sleep Composite x CSF Biomarker Status			0.07 (0.09)

R^2^	0.11	0.16	0.12
Adjusted R^2^	0.10	0.14	0.09

* p < 0.05

** p < 0.01

*** p < 0.001.

**Table 5:** Linear mixed-effects models predicting daily cognition. CSF positivity was defined as CSF p-tau181/Aβ42 > 0.0649.

Covariate	Model 1 – Full Sample	Model 2 – Full Sample; *APOE* ε4 Interaction	Model 3 - Subsample; CSF Biomarker Interaction
	*b (SE)*	*b (SE)*	*b (SE)*
Age (z-scored years)	0.15 (0.03)	0.17 (0.03) ***	0.17 (0.03) ***
Gender (Male)	0.09 (0.05)	0.07 (0.05)	0.08 (0.08)
Education (z-scored years)	−0.06 (0.03) *	−0.05 (0.03) *	0.03 (0.04)
Sleep Composite	0.01 (0.01)	0.01 (0.01)	0.01 (0.01)
Day’s Completed	−0.07 (0.02) **	0.06 (0.02) **	0.10 (0.03) **
*APOE* ε4 Status (Positive)		0.2 (0.07) **	
Sleep Composite x *APOE* ε4 Status		0.00 (0.02)	
CSF Biomarker Status (Positive)			0.07 (0.12)
Sleep Composite x CSF Biomarker Status			0.03 (0.03)

Observations	1890	1885	1214

* p < 0.05

** p < 0.01

*** p < 0.001.

**Table 6:** Generalized additive models predicting cognition. CSF positivity was defined as CSF p-tau181/Ap42 > 0.0649. EDF = Effective Degrees of Freedom, Ref. df = Reference Degrees of Freedom.

Covariate	Model 1 – Full Sample	Model 2 – Full Sample; *APOE* ε4 Interaction	Model 3 - Subsample; CSF Biomarker Interaction
	*b (SE)*	*b (SE)*	*b (SE)*

Age (z-scored years)	0.17 (0.01) ***	0.18 (0.01) ***	0.17 (0.02) ***
Gender (Male)	0.06 (0.02) **	0.04 (0.02)	0.06 (0.03)
Education (z-scored years)	0.05 (0.01) ***	0.04 (0.01) **	0.01 (0.02)
Day’s Completed	0.06 (0.01)	0.06 (0.01) ***	0.08 (0.02) ***
*APOE* ε4 Status (Positive)		0.19 (0.02) ***	
CSF Biomarker Status (Positive)			0.18 (0.04) ***

Spline Fit	EDF (Ref. df)	EDF (Ref. df)	EDF (Ref. df)

Sleep Composite Deviation	2.4 (2.7) *		
Sleep Composite Deviation x *APOE* ε4 Status (Positive)		2.1 (2.5)	
Sleep Composite Deviation x *APOE* ε4 Status (Negative)		1.9 (2.4)	
Sleep Composite Deviation x CSF Biomarker Status (Positive)			1.00 (1.00)
Sleep Composite Deviation x CSF Biomarker Status (Negative)			1.5 (1.9)
Participant ID	0.94 (1.0)	0.94 (1.0) ***	0.94 (1.0) ***

Observations	1890	1885	1214

* p < 0.05

** p < 0.01

*** p < 0.001.
